# FastMM: an efficient toolbox for personalized constraint-based metabolic modeling

**DOI:** 10.1186/s12859-020-3410-4

**Published:** 2020-02-21

**Authors:** Gong-Hua Li, Shaoxing Dai, Feifei Han, Wenxing Li, Jingfei Huang, Wenzhong Xiao

**Affiliations:** 1 State Key Laboratory of Genetic Resources and Evolution, Kunming Institute of Zoology, Chinese Academy of Sciences, Kunming, 650223 Yunnan China; 2 Immue and Metabolic Computational Center, Massachusetts General Hospital, Harvard Medical School, Boston, MA 02114 USA; 3 Collaborative Innovation Center for Natural Products and Biological Drugs of Yunnan, Kunming, 650223 Yunnan China; 4 Stanford Genome Technology Center, Stanford University, Palo Alto, CA 94304 USA

**Keywords:** FastMM, Constraint-based model, Metabolic modeling, Metabolism

## Abstract

**Background:**

Constraint-based metabolic modeling has been applied to understand metabolism related disease mechanisms, to predict potential new drug targets and anti-metabolites, and to identify biomarkers of complex diseases. Although the state-of-art modeling toolbox, COBRA 3.0, is powerful, it requires substantial computing time conducting flux balance analysis, knockout analysis, and Markov Chain Monte Carlo (MCMC) sampling, which may limit its application in large scale genome-wide analysis.

**Results:**

Here, we rewrote the underlying code of COBRA 3.0 using C/C++, and developed a toolbox, termed FastMM, to effectively conduct constraint-based metabolic modeling. The results showed that FastMM is 2~400 times faster than COBRA 3.0 in performing flux balance analysis and knockout analysis and returns consistent outputs. When applied to MCMC sampling, FastMM is 8 times faster than COBRA 3.0. FastMM is also faster than some efficient metabolic modeling applications, such as Cobrapy and Fast-SL. In addition, we developed a Matlab/Octave interface for fast metabolic modeling. This interface was fully compatible with COBRA 3.0, enabling users to easily perform complex applications for metabolic modeling. For example, users who do not have deep constraint-based metabolic model knowledge can just type one command in Matlab/Octave to perform personalized metabolic modeling. Users can also use the advance and multiple threading parameters for complex metabolic modeling. Thus, we provided an efficient and user-friendly solution to perform large scale genome-wide metabolic modeling. For example, FastMM can be applied to the modeling of individual cancer metabolic profiles of hundreds to thousands of samples in the Cancer Genome Atlas (TCGA).

**Conclusion:**

FastMM is an efficient and user-friendly toolbox for large-scale personalized constraint-based metabolic modeling. It can serve as a complementary and invaluable improvement to the existing functionalities in COBRA 3.0. FastMM is under GPL license and can be freely available at GitHub site: https://github.com/GonghuaLi/FastMM.

## Background

Constraint-based metabolic models have been developed for over 30 years [1, 2]. As one of the most popular and state-of-art toolbox, COBRA 3.0 [3, 4] can be used to solve a variety of biomedical problems among which are: 1) understanding metabolism related disease mechanisms by Markov Chain Monte Carlo (MCMC) sampling. 2) inferring new potential drug targets by single or multiple gene knockout analysis [5], 3) inferring potential biomarkers by flux variability analysis (FVA) [6], 4) designing anti-metabolites by single or multiple metabolite knockout analysis [7]. The rapid accumulation of genomic and metabolomic data from disease studies, such as TCGA [8], provides an unprecedented opportunity for personalized metabolic modeling of large number of patient samples.

However, three core applications in COBRA 3.0, including MCMC sampling, FVA, and whole genome knockout, require significant time-consuming computing [3, 9]. For example, the genome wide double gene knockouts of human metabolic model require computing time of more than one day on a computer sever. Recently, several applications have been developed to efficiently perform metabolic modeling, for example, Cobrapy to perform constraint-based metabolic modeling for python [9], fastFVA to implement efficient flux variability analysis [10], SL-finder [11] and Fast-SL [12] to conduct genome-wide gene knockout analysis. However, the time cost of metabolic modeling still limits the applications of constraint-based metabolic models to large scale studies.

Here, we developed a toolbox, named FastMM, to implement genome-wide personalized analysis of constraint-based metabolic models. The underlying code of FastMM is written in C/C++ and uses GNU Linear Programming Kit (GLPK) and Gurobi to perform flux balance analysis (FBA) in the constraint-based models. FastMM is 2~400 times faster than COBRA 3.0 and returns consistent results. It serves as a valuable tool for personalized genome-scale metabolic modeling in large disease studies.

## Implementation

### Efficient flux variability analysis and knockout analysis

The underlying code of flux variability analysis and knockout analysis in FastMM were written in C/C++, and included six core modules: FBA, FVA, singleGeneKO, doubleGeneKO, singleMetKO, and doubleMetKO, which represent flux balance analysis, flux variability analysis, genome wide single gene knockout analysis, genome wide double gene knockout analysis, genome wide single metabolite knockout analysis, and genome wide double metabolite knockout analysis, respectively. All the six core programs call GLPK or Gurobi solver and use a two-phase primal simplex method to iteratively solve the following standard FBA linear programming (LP):

1 $$ {\displaystyle \begin{array}{c}\mathit{\max}\ \mathrm{or}\;\mathit{\min}\;{C}^TV\\ {}\mathrm{Subject}\ \mathrm{to}:\mathrm{S}\times \mathrm{V}=0\\ {}\mathrm{Vl}<V<\mathrm{Vu}\end{array}} $$

Where S is the stoichiometry matrix with m rows and n columns. m and n are the number of metabolites and reactions, respectively. *C*^*T*^ is a vector of the coefficient factors of the objective function(s). In most cases, the vector just contains one non-zero element. V_l_ and V_u_ is the lower and upper bounds of the flux. For knockout analysis, V_l_ and V_u_ can be different from wide type when there is a gene or a metabolite knockout.

To perform efficient knockout analysis, we employed an algorithm to reduce the number of LPs, which was similar as Fast-SL [12]. Firstly, we solved a LP to minimize the sum of reaction fluxes while the wild type objective function (*C*^*T*^*V*) was optimized, termed as *f*, this LP can be written as following:

2 $$ {\displaystyle \begin{array}{c}\kern6em \min \sum \mid \mathrm{V}\mid \\ {}\mathrm{S}\mathrm{ubject}\ \mathrm{to}:\kern2em \mathrm{S}\times \mathrm{V}=0\\ {}\kern7em \mathrm{Vl}<V<\mathrm{Vu}\\ {}\kern6em {\mathrm{C}}^TV=f\end{array}} $$

Then, we can obtain a small set of non-zero flux reactions set *J* in the meanwhile the wild-type objective function (*C*^*T*^*V*) was optimized. In knockout analysis, only the genes (or metabolites), taking participated in set *J* reactions, were used to perform further knock out analysis. Other genes (or metabolites) will be considered as invalid since they do not affect the wild-type objective function (*C*^*T*^*V*). For example, when we used this algorithm to the consistent general human metabolic model *consistRecon2_v3* (including 5317 reactions, 2960 metabolites and 2194 genes) [13, 14], and set the “biomass_reaction” as the wild-type objective function, we obtained 245 non-zero flux reactions, and only 251 genes take participant in these reactions. In the case of double gene knockout analysis, the number of total LPs was greatly reduced (from 4.8 × 10^6^) to 63,001.

### Efficient MCMC sampling

The underlying code of MCMC sampling was also written in C/C++, and uses the hit and run MCMC algorithm. The detailed information of this algorithm was well document in COBRA 3.0 [4]. Briefly, FastMM firstly generated the initial warming up points using GLPK or Gurobi solver, then implemented hit-and-run sampling based on the initial warm points. Since the sampling procedure requires intensive linear algebraic computation, we used the state-of-art basic linear algebra subprograms (BLAS) library, known as Intel® Math Kernel Library (Intel® MKL), to perform large-scale MCMC sampling. While the underlying BLAS of MKL is multiple thread, the MCMC is also automatically multiple threaded base on the computer CPU. By default, the number of MCMC threading is the half of the number of CPU.

### Matlab/octave interface and multiple threading

The Matlab interface was developed to ensure FastMM is fully compatible with the COBRA 3.0. The multiple threading of FVA and knock out analysis in FastMM was developed using the Matlab parallel computational toolbox. Users can define the number of CPU in each FastMM Matlab interface function.

### Development of “one-command” protocol

To ensure that FastMM could be easily and correctly used by users without a strong metabolic modeling background, we developed a “one-command” protocol. This protocol firstly reconstructed the tissue-specific metabolic model using the gene (or protein) expression information via the Fastcore method [14] or mCADRE [15]. And then, the flux variability analysis and knockout analysis were conducted by employing the precompiled FastMM core modules. The only input is the gene expression matrix, and all of the results are stored in the. /out subdirectory.

## Results

### Overall of FastMM

FastMM project (https://github.com/GonghuaLi/FastMM) is aimed to provide an efficient, compatible, and user-friendly toolbox/package for personalized constraint-based metabolic modeling. FastMM is under GPL license and located at GitHub in order to allow all developers in this field to contribute. In current version (01/30/2020), FastMM supports three LP solvers (GLPK, Gurobi and Cplex), and contains two layers, including core modules layer and the Matlab interface layer.

The core modules layer of FastMM was written in C/C++, and can be compiled and run in nearly all platforms (such as Windows, Mac-OS, and Linux). This layer contains seven applications: FBA, FVA, singleGeneKO, doubleGeneKO, singleMetKO, doubleMetKO and FastMCMC. FastMM uses small memory (20~30 M for FVA and knockout analysis, and ~ 100 M for MCMC sampling) and can be run on different types of computers, such as PC, Mac, server, and supercomputer.

The Matlab interface layer was developed to make FastMM fully compatible with COBRA 3.0 and user-friendly. This interface layer standardizes input datasets, calls executable files in core modules layer, performs multiple threading and generates outputs.

Taken together, unlike the COBRA toolbox design strategy, FastMM separated the constraint-based metabolic modeling procedures into two layers. All time cost procedures, such as FVA and genome-wide knockout analysis, were written in C/C++, making FastMM efficient. On the contrary, other procedures, including multiple threading, model reconstruction, dataset standardization and input/output generation, were wrapped and written in Matlab/Octave, making FastMM compatible with COBRA 3.0 and user-friendly.

### FastMM is efficient

FastMM is efficient for both flux variability and knockout analysis. We applied FastMM and COBRA 3.0 (using Gurobi solver) to analyze the consistent general human metabolic model Recon 2.03 (5317 reactions and 2960 metabolites) [13, 14] on a CPU of Intel Xeon E5–2640 2.60GHz. COBRA 3.0 has made a large improvement on computational efficiency compared with COBRA 2.0 (Table 1). For flux variability analysis, FastMM and COBRA 3.0 spent 80 s, and 183 s, respectively. For genome wide single gene knockout analysis, FastMM and COBRA 3.0 spent 9 s, and 19 s, respectively. For genome wide double gene knockout analysis, FastMM and COBRA 3.0 spent 260, and 118,570 s, respectively. These results showed that FastMM is 2~400 times faster than COBRA 3.0 (Table 1). The results of FVA and single gene knockout analysis using FastMM are also consistent with the results of COBRA 3.0 (Supplementary Table S1-S3).

**Table 1:** Comparison of the time cost of metabolic modeling between FastMM and other software using Gurobi solver

Applications	COBRA 3.0	Cobrapy	fastFVA	Fast-SL	FastMM
FVA	^$^183 s	99 s	^**^85 s	\	^***^80 s
SingleGeneKO	19 s	15 s	\	21 s	9 s
DoubleGeneKO	118,570 s	^*^2324 s	\	3161 s	260 s
SingleMetKO	\	\	\	\	8 s
DoubleMetKO	\	\	\	\	578 s
MCMC	2185 s	\	\	\	254 s

**Note:** The consistent general human metabolic model Recon 2.03 (5317 reaction, 2960 metabolites, and 2194 genes) was used in the analyses. The symbols of “s” represents seconds. MCMC sampling uses the parameter of points of 2000, and steps of 1000. All FastMM applications used one CPU cores, other software used default parameters. \: not available for the corresponding applications. $: COBRA 3.0 used 4 CPU cores to perform flux variability analysis. *: Cobrapy used 4 CPU cores to perform double gene knockout. ^**^: fastFVA do not support the Gurobi solver, the time cost (85 s) was calculated using GLPK solver. ^***^: FastMM consumed 80 and 107 s to conduct flux variability analysis using Gurobi and GLPK solver, respectively

We also compared FastMM with recently applications for FVA and knockout analysis, including Cobrapy, fastFVA, SL-finder and Fast-SL. When performing FVA, FastMM is slightly faster than Cobrapy (Table 1). Since fastFVA does not support the Gurobi solver, we compared FastMM and fastFVA using GLPK solver, FastMM and fastFVA spent 107 and 85 s respectively, indicating FastMM is slightly slower than fastFVA. However, when using Gurobi solver, FastMM spend 80 s.

For the knockout analysis, FastMM is significantly faster than the state-of-art efficient applications, including Cobrapy and Fast-SL (Table 1). For example, in double gene knockout analysis, FastMM is 9 and 12 times faster than Cobrapy and Fast-SL, respectively. SL-finder was an application to find synthetic lethal pairs [11]. Due to the platform issue (SL-finder was running on General Algebraic Modeling System), we did not directly compare FastMM with SL-finder. However, it was reported that fast-SL is comparable (or similar) efficient with SL-finder [12].

Design of novel drugs using metabolite analogues have been reported as a promising strategy for metabolism-based drug design [7, 16]. Metabolite analogues could competitively bind the enzyme active sites and result to inhibit the corresponding reactions. FastMM provides an efficient solution to find candidate metabolite analogues, while other toolboxes do not (Supplementary Table S4).

FastMM is also efficient for MCMC sampling. We applied FastMM to perform MCMC sampling in consistent Recon 2.03 with the parameters of 2000 points and the 1000 steps. FastMM spent 254 s, while the COBRA 3.0 spent 2185 s. FastMM was 8 times faster than COBRA 3.0 for MCMC sampling. In addition, the MCMC output of FastMM was correct, and the error was small than 1e-7 (Table S5).

FastMM is flexible. Users not only can define one or multiple objective function(s), but also can define additional constraints. For example, users can use singleGeneKO to find which gene knockout changes the status of secretory metabolites (see the user manual in Supplementary Materials).

### FastMM is easy to use

FastMM can be implemented by “one-command” mode. The only requirement for running FastMM is the parameter file. This file defines the following information: i). the path of the gene expression matrix file, ii) the path of objective function(s) file, iii). the cutoff of gene expression, and iv). number of CPU for performing metabolic modeling. After defined these parameters, the users can just type one command in Matlab/Octave:

> > FastMM

Then, all of the metabolic modeling, including FVA, knockout analysis, and MCMC simulation, will be performed, and the results will be stored in the “./out” subdirectory.

### Example and advanced use

**Fig. 1:**
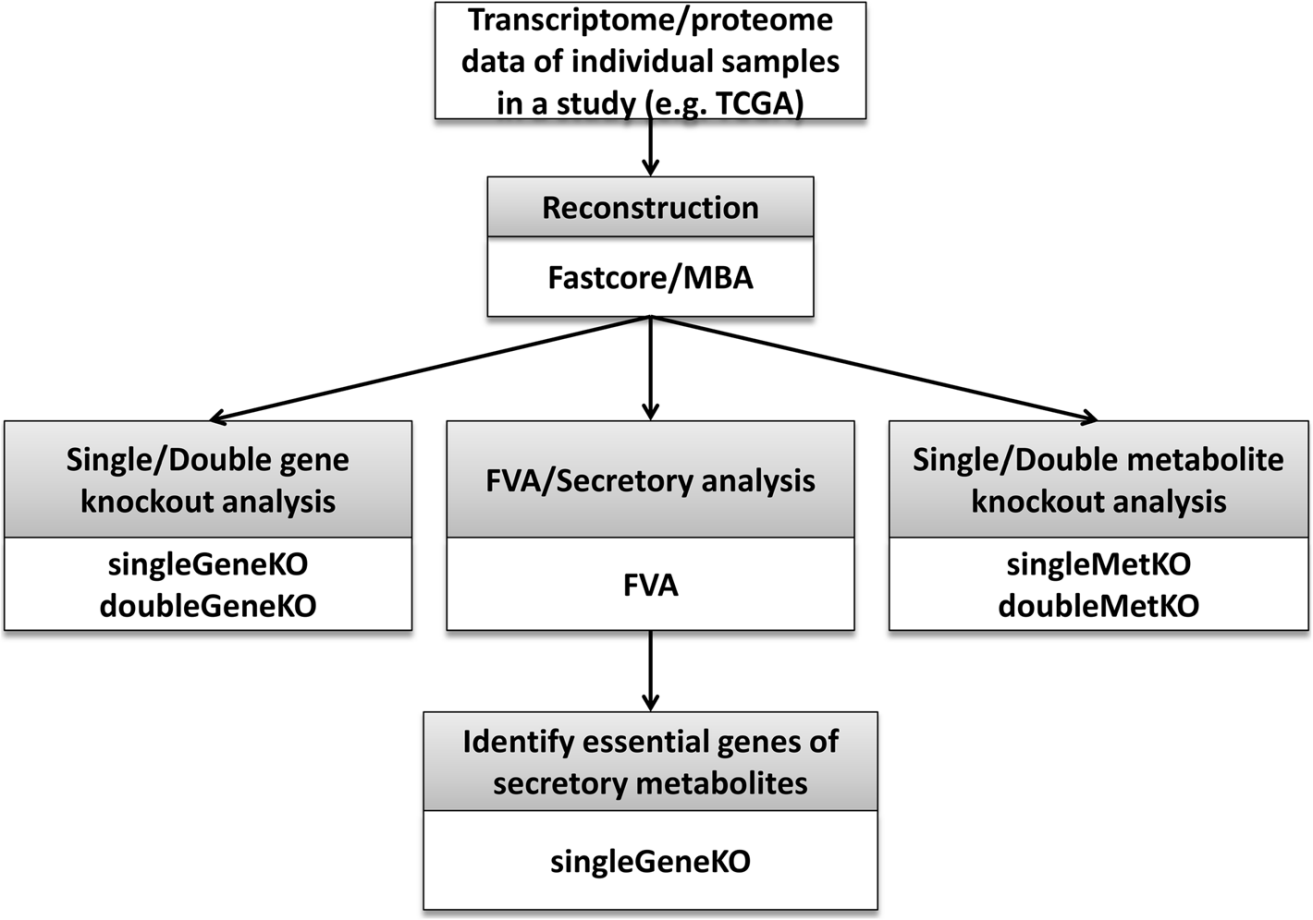
Flowchart of personalized genome-scale knockout analysis of constraint-based metabolic models by FastMM. Shown in gray boxes are the steps of the procedure followed by, in white boxed, the names of the modules of FastMM

Since FastMM is ultra-efficient and user friendly, we can now perform personalized genome wide metabolic modeling in large scale disease studies. As an example, we applied it to 528 individual samples of lung cancer in TCGA to analyze the individual cancer metabolic profiles. The detailed protocol and results are described in the user manual (https://github.com/GonghuaLi/FastMM/tree/master/doc). Briefly, these can be performed by the following five steps (Fig. 1):

i) Model reconstruction. Many methods can be used to reconstruct tissue specific metabolic model, such as MBA [17] and fastcore [14]. In this study, we used fastcore, since it balances between the time cost of computing and accuracy. In this study, we used one of FastMM matlab function, name “reconstruction_by_fastcore.m”, and obtained 528 individual TCGA lung cancer models. There are 58 normal samples and 470 lung cancer samples. In this procedure, we used the cutoff of RESM as 75, because RESM is about 25 times larger than RPKM [18], where RPKM > 3 was consider as expressed or highly expressed [19]. Actually, there are about 10,000~12,000 genes with the RESM > 75, which is consistent with Ramskold’s result [20]. The reconstruction can be implemented using FastMM:

> > *rxnsmatrix = reconstruction_by_fastcore(consmodel, expr, 75)*

Where the Matlab input is the consistent cobra model(consistent recon 2 [14]), the gene expression matrix and the gene expression cutoff. The output is the constructed model with the binary format m × n matrix. m is the number of reactions in consistent input cobra model, n is the number of samples (*n* = 528 in this case).

ii) Genome wide single and double gene knockout analysis to identify individual lethal and synergistic lethal genes. Because the program “doubleGeneKO” contains both single and double gene knockout results, we can implement single and double gene knockout analysis by the following Matlab interface function using 8 threading:

*> > GeneKOout = FastMM_doubleGeneKO_multi(consmodel, rxnsmatrix, 8)*

The results suggested that PEPD, SLC15A1, and SLC5A3 were candidates for individual lethal genes specific to lung cancer, and pairs of CBS-SLC7A11, CBS-SLC3A2, CMPK1-PTDSS1, CMPK1-PLD2 were candidate synergistic lethal genes specific in the tumors. GUK1 is a target for a number of cancer chemotherapeutic agents [21]; however among the 528 TCGA samples, while deletions of GUK1 was lethal in 93% of the normal controls, it was not lethal in 71% of the lung cancers.

iii) Large scale FVA to infer cancer-specific secretory metabolites. Identifying the cancer-specific secretory metabolites could be help to understand the cancer environment mechanism and environment-based drug design and can be implemented by flux variability analysis. In FastMM, we can perform multiple threading FVA by the following function (eg, using 8 CPU):

*> > [fluxmin,fluxmax] = FastMM_FVA_multi(consmodel,rxnsmatrix,8)*

The results suggested that 16 metabolites were specifically secreted in the tumors of lung cancer (5%~ 19%).

iv) Genome-wide gene knockout analysis to identify genes that affect cancer cell secretion. After the identification of the cancer-specific secretory metabolites, we need to know which genes affect the secretion of these metabolite. This procedure could be performed by gene knock out analysis using the user defined objective functions. Here is the Matlab interface function (using 8 threading):

> > *Out = FastMM_singleGeneKO_multi(consmodel,rxnsmatrix,8,’-f secret_rxns.txt’)*

Where “secret_rxns.txt” is the user-defined objective function file, containing 55 exchange reactions from last step. The result indicated that 97 genes would be essential for metabolite secretion only in tumor samples. Knockout of these genes, such as SLC35D1 and UGCG, would not directly affect the cancer cell growth, but could alter the micro-environment of cancer cells.

v) Genome wide single and double metabolite knockout analysis to predict lethal and synergistic lethal metabolites. Anti-metabolite drug design is one of the promise anti-cancer strategy [7]. Identifying which metabolite is essential for cancer growth but not affect the normal cells could provide novel anti-cancer metabolite targets [7]. This procedure can be performed by metabolic knockout analysis. Using FastMM, we can just type one command in the Matlab (using 8 threading):

> > metout = FastMM_doubleMetKO_multi(model, rxnsmatrix,8)

The result suggested that the pair of AMP and 5-Phosphoribosyl diphosphate (PRPP) is synergistic lethal in 93% of the controls but only 29% tumors, suggesting that alternative metabolic pathways are activated in the majority of the tumor samples (71%).

## Discussion

In the past decades, along with the high throughput of “omic” technologies developed, large-scale biochemical and clinical datasets have been available and give us the opportunity to find novel metabolic mechanisms and novel metabolic targets in individual patients. Several toolbox, such as COBRA toolbox [3, 22], RAVEN [23], have been developed to perform metabolic modeling to study metabolic dysfunction in various diseases [5, 17, 24], however, the computational time still limits the large scale application.

In this study, we developed a novel toolbox to efficiently perform metabolic modeling. FastMM is 3~400 times faster than COBRA 3.0 in flux variability analysis and knockout analysis and returned the consistent results. FastMM also had 8 times faster than COBRA in MCMC sampling. FastMM is also faster than the most of other efficient metabolic modeling applications, such as Cobrapy [9] and Fast-SL [12]. Thus, FastMM covered the computation time limitation and can be used in large-scale metabolic modeling.

For flux variability analysis, the state-of-art efficient software is fastFVA [10]. Similar as FastMM, the underlying code of fastFVA was also written in C/C++, thus fastFVA and FastMM FVA have similar efficient. FastMM FVA supports both Gurobi and Cplex solvers while fastFVA only supports cplex solver.

For knock out analysis, FastMM is significantly faster than nearly all state-of-art efficient software. For example, in the case of double gene knockout analysis, FastMM is 8 and 12 times faster than Cobrapy [9] and Fast-SL [12]. The ultra-efficiency of FastMM come from the employment of a algorithm similar to Fast-SL [12] to significantly reduce the number of total LPs. Unlike SL-finder [11] and Fast-SL [12], the current version (01/31/2020) of FastMM do not support the triple and other high level knockout analysis. Besides gene knockout analysis, FastMM also provides efficient solution for metabolite knockout analysis, which would help to accelerate the anti-metabolite drug design [16].

We also wrote a large number of Matlab interface functions to make sure FastMM can fully compatible with the state-of-art metabolic modeling toolbox COBRA 3.0. These Matlab functions not only provide a “one-command” metabolic modeling solution for little background users, but also provide advanced and multiple threading solution for users with strong programming background users.

There is still a limitation in FastMM. In this study, we used the Fasctcore [14] as the example. Unfortunately, nearly all published methods, such as MBA [17], Fastcore [14], GIMME [25], just use the Presence/Absence call of gene expression or protein abundance to perform tissue specific metabolic modeling [26]. Most of quantitative expression information was lost in reconstruction and would result in fail to identify the metabolic changes. Besides, in recent FastMM release, only Matlab interface was developed, this would be not convenience for the users without Matlab license. We would develop python interface of FastMM in next release.

## Conclusion

FastMM is an efficient toolbox for metabolic modeling in large scale disease studies. FastMM enables the identification of personalized targets of genes and metabolites as new candidates for therapy or biomarkers, by personalized metabolic modeling on each of the hundreds to thousands of samples in a disease study.

## Availability and requirements

Project name: FastMM

Project home page: https://github.com/GonghuaLi/FastMM

Operating system(s): Platform independent

Programming language: MATLAB and C

Other requirements: COBRA toolbox, Gurobi 5.0 or higer, and Cplex 12.0 or higher

License: GNU AGPLv3

Any restrictions to use by non-academics: license needed

## Supplementary information

**Additional file 1 Table S1.** Comparison of the time cost of metabolic modeling between FastMM and other software using Gurobi solver.

**Additional file 2 Table S2.** Single gene knockout results between COBRA 3.0 and FastMM.

**Additional file 3 Table S3.** FVA results between COBRA 3.0 and FASTMM.

**Additional file 4 Table S4.** Single knockout results of 2960 metabolites in FastMM.

**Additional file 5 Table S5.** Maximum MCMC error using FastMM and COBRA 3.0.

## Data Availability

The software and related data can be found at https://github.com/GonghuaLi/FastMM. The datasets supporting the conclusions of this article are included within article (and its Additional files).

## Abbreviations

FBA: flux balance analysis
FVA: flux variability analysis
MCMC: Markov Chain Monte Carlo
