# Correction to: FastMM: an efficient toolbox for personalized constraint-based metabolic modeling

**DOI:** 10.1186/s12859-020-03723-y

**Published:** 2020-09-16

**Authors:** Gong-Hua Li, Shaoxing Dai, Feifei Han, Wenxing Li, Jingfei Huang, Wenzhong Xiao

**Affiliations:** 1 State Key Laboratory of Genetic Resources and Evolution, Kunming Institute of Zoology, Chinese Academy of Sciences, Kunming, 650223 Yunnan China; 2 Immue and Metabolic Computational Center, Massachusetts General Hospital, Harvard Medical School, Boston, MA 02114 USA; 3 Collaborative Innovation Center for Natural Products and Biological Drugs of Yunnan, Kunming, 650223 Yunnan China; 4 Stanford Genome Technology Center, Stanford University, Palo Alto, CA 94304 USA

**Correction to: BMC Bioinformatics 21, 67 (2020)**

**https://doi.org/10.1186/s12859-020-3410-4**

Following publication of the original article [1], the authors identified an error in the author name of Wenxing Li.

1. Incorrect name:

Wenxin Li

2. Correct name:

Wenxing Li

The author group has been updated above and the original article [1] has been corrected.
